# An ATP-responsive metal–organic framework against periodontitis via synergistic ion-interference-mediated pyroptosis

**DOI:** 10.1093/nsr/nwae225

**Published:** 2024-06-26

**Authors:** Qijing Yang, Xiaolin Sun, Qihang Ding, Manlin Qi, Chengyu Liu, Tingxuan Li, Fangyu Shi, Lin Wang, Chunyan Li, Jong Seung Kim

**Affiliations:** Department of Prosthodontics, Jilin Provincial Engineering Laboratory of Intelligent Oral Treatment Technology, School and Hospital of Stomatology, Jilin University, Changchun 130021, China; Department of Prosthodontics, Jilin Provincial Engineering Laboratory of Intelligent Oral Treatment Technology, School and Hospital of Stomatology, Jilin University, Changchun 130021, China; Department of Prosthodontics, Jilin Provincial Engineering Laboratory of Intelligent Oral Treatment Technology, School and Hospital of Stomatology, Jilin University, Changchun 130021, China; Department of Chemistry, Korea University, Seoul 02841, South Korea; Department of Prosthodontics, Jilin Provincial Engineering Laboratory of Intelligent Oral Treatment Technology, School and Hospital of Stomatology, Jilin University, Changchun 130021, China; Department of Prosthodontics, Jilin Provincial Engineering Laboratory of Intelligent Oral Treatment Technology, School and Hospital of Stomatology, Jilin University, Changchun 130021, China; Department of Prosthodontics, Jilin Provincial Engineering Laboratory of Intelligent Oral Treatment Technology, School and Hospital of Stomatology, Jilin University, Changchun 130021, China; Department of Prosthodontics, Jilin Provincial Engineering Laboratory of Intelligent Oral Treatment Technology, School and Hospital of Stomatology, Jilin University, Changchun 130021, China; Department of Prosthodontics, Jilin Provincial Engineering Laboratory of Intelligent Oral Treatment Technology, School and Hospital of Stomatology, Jilin University, Changchun 130021, China; Department of Prosthodontics, Jilin Provincial Engineering Laboratory of Intelligent Oral Treatment Technology, School and Hospital of Stomatology, Jilin University, Changchun 130021, China; Department of Chemistry, Korea University, Seoul 02841, South Korea

**Keywords:** metal–organic framework, periodontitis, pyroptosis, immunotherapy, ion-interference therapy

## Abstract

Periodontitis involves hyperactivated stromal cells that recruit immune cells, exacerbating inflammation. This study presents an ATP-responsive metal–organic framework (Mg/Zn-MOF) designed for periodontitis treatment, utilizing ion interference to modulate immune responses and prevent tissue destruction. Addressing the challenges of synergistic ion effects and targeted delivery faced by traditional immunomodulatory nanomaterials, the Mg/Zn-MOF system is activated by extracellular ATP—a pivotal molecule in periodontitis pathology—ensuring targeted ion release. Magnesium and zinc ions released from the framework synergistically inhibit membrane pore formation by attenuating Gasdermin D (GSDMD) expression and activation. This action curtails pyroptosis, lactate dehydrogenase and IL-1β release, thwarting the onset of inflammatory cascades. Mechanistically, Mg/Zn-MOF intervenes in both the NLRP3/Caspase-1/GSDMD and Caspase-11/GSDMD pathways to mitigate pyroptosis. *In vivo* assessments confirm its effectiveness in diminishing inflammatory cell infiltration and preserving collagen integrity, thereby safeguarding against periodontal tissue damage and bone loss. This investigation highlights the promise of ion-interference strategies in periodontitis immunotherapy, representing a significant stride in developing targeted therapeutic approaches.

## Teaser

An ATP-responsive metal–organic framework was developed for periodontitis immunotherapy, enabling targeted ion delivery to the inflamed site and facilitating efficient ion-interference therapy to suppress pyroptosis and safeguard periodontal tissues.

## INTRODUCTION

Periodontitis—a chronic inflammatory disease triggered by microbial dysbiosis—is characterized by the progressive destruction of periodontal supporting tissues, initiated by plaque accumulation [1]. It is considered to be both the leading cause of tooth loss in adults and a risk factor for a variety of systemic diseases, such as diabetes and Alzheimer's disease [2–4]. The consensus in current research is that disruption of immune homeostasis is a critical factor in both the onset and development of periodontitis. Therefore, more attention has been drawn to alleviating inflammation destruction by modulating the immune response as a therapeutic strategy for periodontitis.

Pyroptosis—an inflammatory form of programmed cell death—is governed by the Gasdermin superfamily of proteins and occurs via two distinct pathways: the canonical pathway, involving Caspase-1, and the non-canonical pathway, involving Caspase-4/5/11, which are activated by virulence factors, such as lipopolysaccharide (LPS) [5,6]. Recent studies have increasingly demonstrated a strong association between pyroptosis and periodontitis [7–10]. Certain virulence factors trigger inflammasome activation, precipitating pyroptosis and consequently alveolar bone loss [11]. Hence, pyroptosis presents a viable target for periodontal immunotherapy.

Bioactive ions are instrumental in numerous physiological and biochemical reactions. Aberrant distributions of these ions within and outside cells can activate signaling pathways, affect protein or enzyme configurations and potentially lead to cellular damage or death. The therapeutic strategy that harnesses the properties of bioactive ions is referred to as ion-interference therapy. Initially proposed for tumor treatment, this approach includes strategies such as Ca^2+^ overload and copper-ion-interference therapy [12,13]. Given the ubiquitous presence of these functional ions in most cells and their role in regulating diverse signaling pathways, ion-interference therapy theoretically offers universal applicability for addressing a broad spectrum of diseases. Moreover, the metallic ions used in ion-interference therapy are essential elements of organisms and demonstrate excellent biocompatibility. Consequently, bio-responsive nanocomposites designed for ion-interference therapy show significant clinical applicability and translational potential. Notably, ion therapy involving copper and iron ions has been employed in the antibacterial treatment of periodontitis [14]. However, further research is necessary to fully explore the potential of ion-interference therapy in modulating immunity against periodontitis. Ion-interference-based immunotherapy, which modulates pyroptosis, has been noticed for its high efficiency, minimal side effects and reduced risk of drug resistance [15,16]. Evidence indicates that Mg^2+^ inhibits pyroptosis by blocking Ca^2+^ influx, thereby preventing the assembly and membrane localization of Gasdermin D N-terminal (GSDMD-NT) upon activation [17]. Furthermore, *Porphyromonas gingivalis*—a key pathogen in periodontitis—can initiate the non-canonical pyroptosis pathway through its outer membrane vesicles and LPS [18]. Zn^2+^, as established in prior research, effectively hinders the activation of Caspase-11 by impairing signaling via a toll/interleukin-1 receptor domain containing adaptor-inducing interferon β–interferon regulatory factor 3–signal transducer and activator of transcription factor 1 (TRIF-IRF3-STAT1), reducing cellular response to LPS and potentially blocking the non-canonical pyroptosis pathway at its inception [19]. Integrative mechanistic analyses advocate that ion-interference-based immunotherapy could significantly mitigate the inflammatory response and immune hyperactivity instigated by pyroptosis in periodontitis. Current ion delivery methodologies include ion-doped bioactive glass, microneedles, microspheres, hydrogels, nanoparticles and metal–organic frameworks (MOFs) tailored for specific application scenarios [20–23]. However, research on ion modulators frequently overlooks the synergistic potential of ions, focusing on singular ions instead. Additionally, achieving targeted delivery to infection sites is essential for effectively counteracting pyroptotic activity.

Recently, MOFs—a novel class of coordination compounds composed of organic ligands and metal ions/ion clusters interconnected through coordination bonds—have emerged as a topic of interest in biomedicine for their exceptional capacity to store and release metal ions with high efficiency. These MOFs are frequently engineered to integrate bioactive metal cations (Fe^2+/3+^, Ca^2+^, Cu^2+^, Zn^2+^) that are released upon framework degradation, playing essential roles in biological systems [24–27]. The inherent porous architecture of MOFs facilitates the diffusion of stimulus-responsive guest molecules, endowing these materials with the ability to react dynamically to external stimuli—a property that is highly beneficial for various biomedical applications [28]. Among the MOFs, the zeolitic imidazolate framework-8 (ZIF-8) consisting of Zn^2+^ and 2-methylimidazole ligands stands out for its straightforward synthesis, high surface area, remarkable ion storage capacity, sensitivity to pH and adenosine triphosphate (ATP), alongside its favorable biocompatibility [29].

In periodontitis, pyroptotic cells release damage-associated molecular patterns such as ATP, which in turn can trigger further pyroptosis via the NLRP3/Caspase-1/GSDMD pathway, perpetuating a detrimental feedback loop of ‘pyroptosis–ATP release–pyroptosis’ [30]. ATP—a critical signaling molecule in periodontitis—can chelate with zinc ions through its adenine nitrogen and phosphate groups. This interaction underpins the ATP-responsive behavior of ZIF-8, making it exceptionally well suited for applications in periodontitis [31]. In the molecular structure of ZIF-8, Zn^2+^ forms a tetrahedral coordination complex with the nitrogen atom of the 2-methylimidazole, while also offering vacant orbitals that can engage in additional coordination bonds. Analogously, the natural structure of chlorophyll with Mg^2+^ suggests that a similar tetrahedral coordination is feasible with a nitrogen atom, leveraging the unoccupied orbitals of Mg^2+^ [32]. Therefore, the strategic design of bimetallic MOFs, which incorporate Mg^2+^ and Zn^2+^ within a ZIF-8-like framework, holds the potential to enhance targeted response and synergistic ion delivery, ultimately advancing periodontitis immunotherapy by modulating pyroptosis.

**Scheme 1.**
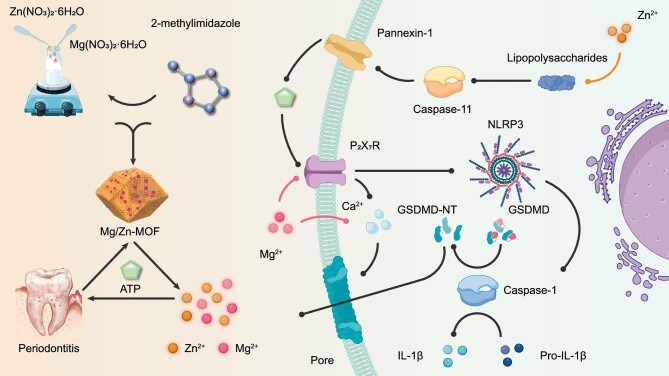
Synthesis and application of Mg/Zn-MOF nanocomposite for periodontitis treatment. In inflamed tissues, under the influence of elevated ATP levels, Mg/Zn-MOF undergoes decomposition and releases Zn^2+^ and Mg^2+^, which inhibits pyroptosis through both canonical and non-canonical pathways, ultimately mitigating periodontal tissue damage.

In this study, we present a bimetallic MOF approach capable of responding to ATP in the periodontal immune microenvironment and suppressing pyroptosis through the release of Zn^2+^ and Mg^2+^ from a Mg/Zn bimetallic MOF (Scheme 1). The Mg/Zn-MOF was synthesized using a single-step solvothermal method, with 2-methylimidazole employed as an organic ligand. This framework preferentially binds ATP over 2-methylimidazole due to the stronger affinity between ATP and Zn^2+^, leading to the ATP-sensitive behavior of the MOF. This property enables Mg/Zn-MOF to undergo precise decomposition at sites of inflammation where ATP levels are high, while maintaining relative stability under normal physiological conditions. This selective behavior is crucial for the targeted delivery and controlled release of Zn^2+^ and Mg^2+^, thereby enhancing ion utilization and mitigating side effects caused by fluid diffusion compared with ion application alone. The Mg/Zn-MOF is designed to regulate GSDMD to prevent the formation of membrane pores through both the NLRP3/Caspase-1/GSDMD and Caspase-11/GSDMD pathways, effectively curtailing pyroptosis. Therefore, the Mg/Zn-MOF offers a therapeutic strategy for alleviating periodontal tissue damage and the associated inflammatory response by regulating pyroptosis. This innovative nanocomposite shows great promise for the treatment of periodontitis and suggests a novel direction for the immunotherapy of inflammatory diseases, with substantial clinical application potential.

## RESULTS AND DISCUSSION

### Characterization of Mg/Zn-MOF

**Figure 1.**
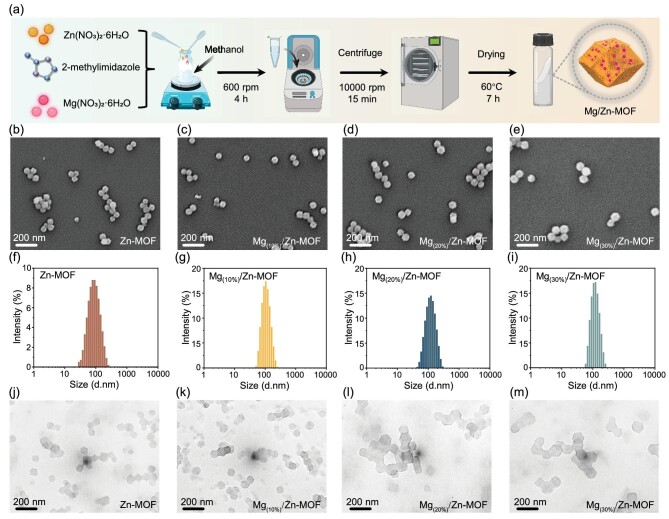
Morphology and particle size distribution of nanocomposites. (a) Flowchart of material synthesis. (b–e) Representative SEM images of (b) Zn-MOF, (c) Mg_(10%)_/Zn-MOF, (d) Mg_(20%)_/Zn-MOF, (e) Mg_(30%)_/Zn-MOF. (f–i) Size distribution of various nanocomposites detected by using the DLS technique. (j–m) Typical TEM images of (j) Zn-MOF, (k) Mg_(10%)_/Zn-MOF, (l) Mg_(20%)_/Zn-MOF, (m) Mg_(30%)_/Zn-MOF.

Mg/Zn-MOF was synthesized via the solvothermal method in a methanol system, utilizing zinc nitrate hexahydrate and magnesium nitrate hexahydrate as metal sources, with 2-methylimidazole serving as the organic ligand (Fig. 1a). Scanning electron microscopy (SEM) revealed that both Zn-MOF and Mg/Zn-MOF exhibited a consistent rhombus dodecahedron structure (Fig. 1b–e). Transmission electron microscopy (TEM) observations and dynamic light scattering (DLS) measurements confirmed that the nanoparticle size gradually increased with higher Mg^2+^ doping ratios (Fig. 1f–m). The mean diameters of Zn-MOF, Mg_(10%)_/Zn-MOF, Mg_(20%)_/Zn-MOF and Mg_(30%)_/Zn-MOF were measured at 85.1, 102.8, 108.6 and 113.5 nm, respectively. Despite the increase in nanoparticle size with higher Mg/Zn ratios, the geometry of the original Zn-MOF structure remained unchanged. It is established that the crystal size of bimetallic MOFs depends on the bimetallic composition, maintaining a constant total ratio of metal nodes to ligands [33]. Literature suggests that the increase in crystal size observed in bimetallic ZIF-8, resulting from the reduction in Zn^2+^ metal nodes, is attributable to a slowed nucleation rate upon the introduction of alternative metals [34–36]. Thus, the incorporation of Mg^2+^, reducing Zn^2+^ nodes, is responsible for the observed enlargement in crystal sizes. Furthermore, TEM analysis revealed favorable dispersion of the nanocomposites, while zeta potential data provided additional evidence for the stability between Zn-MOF and Mg/Zn-MOF (Fig. S1).

**Figure 2.**
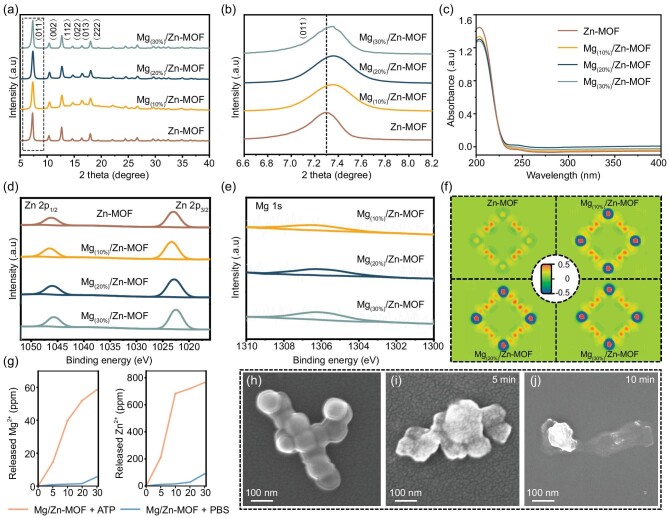
Characterization of nanocomposites. (a) and (b) XRD analysis of Zn-MOF and Mg/Zn-MOF. (c) UV‒vis absorption spectra of various nanocomposites. (d) and (e) XPS analysis of Zn-MOF and Mg/Zn-MOF. (f) Charge density difference diagram of Zn-MOF and Mg/Zn-MOF (red indicates positive charge, blue represents negative charge). (g) Levels of Zn^2+^ and Mg^2+^ released from Mg/Zn-MOF in saline and 5 mM of ATP (*T* = 37°C). (h–j) The SEM images of Mg/Zn-MOF before (h) and after treatment with ATP (5 mM) for (i) 5 min and (j) 10 min.

The examination of Zn-MOF and Mg/Zn-MOF structures through X-ray diffraction (XRD) provided valuable insights into their crystallinity and phase purity (Fig. 2a and b). The XRD patterns of the Zn-MOF closely aligned with the simulated crystal structures of ZIF-8, affirming their structural congruity in terms of unit cells and lattice constants [37]. The absence of impurity phases in Mg/Zn-MOF samples indicates that Mg^2+^ substituted for Zn^2+^ uniformly, preserving the intact Zn-MOF framework. A notable shift in the primary diffraction peaks of Mg/Zn-MOF compared with Zn-MOF suggests the incorporation of Mg^2+^ into the lattice, likely due to the smaller ionic radius of Mg (0.66 Å) compared with Zn (0.74 Å). This substitution, as predicted by using the Bragg equation, results in a contracted lattice constant and shifts the XRD peak toward higher angles. Interestingly, the diffraction peaks of Mg_(30%)_/Zn-MOF and Mg_(20%)_/Zn-MOF showed a similar pattern, indicating a limit to the effective incorporation of Mg^2+^ at higher concentrations.

Density functional theory (DFT) calculations were employed to model the Mg/Zn-MOF structures with different Mg^2+^ doping ratios, further elucidating their lattice parameters (Table S1). These calculations corroborated the XRD findings, showing a decrease in the lattice parameter due to Mg^2+^ substitution, confirming that the smaller ionic radius of Mg^2+^ effectively occupies Zn^2+^ sites within the Mg/Zn-MOF structure. Additionally, ultraviolet–visible (UV-vis) spectrum revealed a dominant absorption peak at 207 nm for Mg/Zn-MOF, corresponding to the characteristic peak of Zn-MOF (Fig. 2c), indicating that Mg^2+^ incorporation does not significantly alter the fundamental structure of Zn-MOF [38].

The chemical composition and structural integrity of Zn-MOF and Mg/Zn-MOF were elucidated through X-ray photoelectron spectroscopy (XPS) analysis. Characteristic Zn 2p core-level spectra revealed two symmetric peaks for both Zn 2p_3/2_ and Zn 2p_1/2_ in all nanocomposites (Fig. 2d). Notably, the spectrum of Mg/Zn-MOF shifted toward lower binding energies, indicating that Mg^2+^ incorporation modified the chemical environment, consequently leading to the chemical shift. This shift, indicative of altered electron density, was further analysed through DFT calculations, which showed a significant increase in the electron density around metal atoms upon Mg^2+^ doping (Fig. 2f). The atomic potential model explains that an elevated outer electron density diminishes binding energy due to an enhanced shielding effect, thus accounting for the observed spectral shift.

Furthermore, the presence of Mg^2+^ peaks at 1036.3 eV confirmed the successful integration of the Mg into the MOF structure, replacing part of the Zn (Fig. 2e). However, the increase in Mg^2+^ content did not match the feed ratio linearly, with the actual ratio of Mg^2+^ ranging from 0.42 to 0.68, as opposed to an exponential increase. Inductively coupled plasma optical emission spectroscopy (ICP–OES) analysis quantified the Mg^2+^ concentrations released from varying Mg/Zn-MOF compositions, revealing measured concentrations of 45.6, 66.3 and 69.8 ppm for Mg_(10%)_/Zn-MOF, Mg_(20%)_/Zn-MOF and Mg_(30%)_/Zn-MOF, respectively. The actual percentages of Mg content were measured to be 1.88%, 2.73% and 2.87%, as detailed in Table S2. These results confirmed the successful doping of Mg^2+^ into the Zn-MOF structure. Consequently, Mg_(20%)_/Zn-MOF was selected for further study due to its optimal Mg incorporation efficiency.

The stability of these nanocomposites under physiological conditions was investigated by monitoring the degradation behavior over time. A consistent increase in the UV absorption peak of the imidazole group beyond 24 hours of immersion suggested the stability of the nanocomposites (Fig. S2). Moreover, ICP–OES results confirmed the relative stability of Mg/Zn-MOF and its slow-release characteristics in physiological environments, such as saline and artificial saliva (Figs S3 and S4). The degradation kinetics, significantly accelerated by ATP (Fig. 2g), highlight the stronger affinity of ATP for zinc ions compared with 2-methylimidazole. This leads to rapid nanocomposite degradation and enhanced ion release in the presence of ATP. SEM images post-ATP exposure illustrated morphological distortion, indicating responsive degradation to ATP—a crucial feature for targeted periodontitis therapy (Fig. 2h–j).

### Inhibitory effect of Mg/Zn-MOF on pyroptosis

The mouse fibroblasts (L929) pyroptosis model was established to investigate the inhibitory effect of Mg/Zn-MOF on pyroptosis, since pyroptical gingival fibroblasts not only disrupted the structure and integrity of the periodontal tissue, but also triggered an inflammatory cascade reaction and excessive acquired immunity by releasing a substantial amount of pro-inflammatory factors and damage-associated molecular patterns [39–42]. The model was stimulated with LPS and ATP—a scenario reflecting the high ATP levels and the presence of gram-negative bacteria characteristic of periodontitis—to induce pyroptosis, elevating the GSDMD and IL-1β levels [43,44]. After a 6-hour LPS stimulation, significant increases in GSDMD and IL-1β mRNA expression were observed, indicating pyroptosis onset (Fig. S5). The optimized treatment protocol involved 1 μg mL^−1^ of LPS for 6 hours followed by 5 mM of ATP for 1 hour.

**Figure 3.**
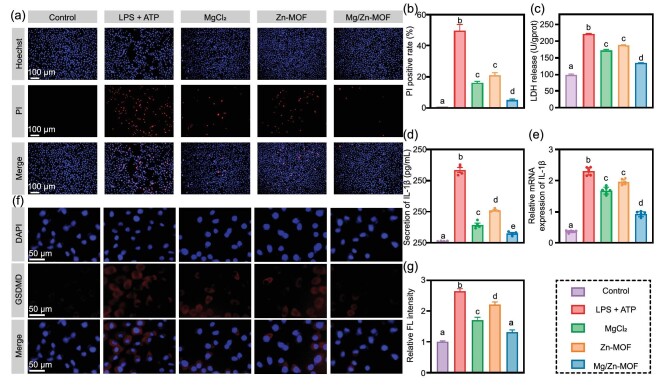
Mg/Zn-MOF attenuates cellular pyroptosis. (a) Representative images of L929 cells stained with Hoechst 33342 (blue) and PI (red). (b) Quantitative assessment of PI-positive cells. (c) The concentration of LDH in the cellular supernatant. (d) The IL-1β concentration in the cell supernatant is assessed by using enzyme-linked immunosorbent assay. (e) Reversed transcription–quantitative polymerase chain reaction (RT–qPCR) results of IL-1β. (f) Representative immunofluorescence images of GSDMD expression in L929 cells. (g) Quantitative analysis of immunofluorescence intensity. (*n* = 6, *P* < 0.05, error bars indicate means ± standard deviations, and different alphabets in bar charts indicate statistically significant differences between the two groups).

Membrane pore formation—a hallmark of pyroptical cells—results in cytoplasmic content release and propidium iodide (PI) uptake [45]. In this study, Hoechst 33342/PI fluorescent staining distinguished between pyroptotic and intact cells. Hoechst 33324-stained nuclei appeared blue, while PI-stained pyroptotic or necrotic cells appear red. Figure 3a and b shows that the PI uptake significantly differs between pretreatment groups, with the LPS + ATP group displaying a high number of red-stained cells, confirming successful pyroptosis induction. Groups treated with MgCl_2_, Zn-MOF and Mg/Zn-MOF exhibited a sequential reduction in PI-labeled cells, indicating pyroptosis inhibition by the Mg and Zn ions, with the most pronounced decrease seen in the Mg/Zn-MOF group due to the synergistic ion effect. Notably, the positive rate of PI uptake was lower for Mg_(20%)_/Zn-MOF compared with Mg_(10%)_/Zn-MOF, suggesting that Mg_(20%)_/Zn-MOF was chosen as the representative Mg/Zn-MOF for enhanced inhibition of pyroptosis (Fig. S6).

Lactate dehydrogenase (LDH) assays further showed the cell membrane integrity, corroborating the staining results. LDH release escalated with LPS + ATP exposure but was notably reduced following MgCl_2_, Zn-MOF or Mg/Zn-MOF treatment, with the lowest levels observed in the Mg/Zn-MOF group, suggesting substantial pyroptosis mitigation (Fig. 3c). Additionally, IL-1β quantification in cell supernatants—an indicator of pyroptosis via inflammasome-activated membrane pores—decreased comparably in the Mg/Zn-MOF group (Fig. 3d). This trend was mirrored in IL-1β mRNA expression (Fig. 3e). GSDMD—a pivotal inflammasome component and executor of pyroptosis—was visualized through immunofluorescence staining, revealing diminished red fluorescence post-treatment in MgCl_2_, Zn-MOF or Mg/Zn-MOF, with the Mg/Zn-MOF group approaching the control level, indicating inhibited pore formation (Fig. 3f and g). Collectively, these findings suggest that Mg/Zn-MOF can attenuate cell pyroptosis by regulating GSDMD to inhibit membrane pore creation.

### Potential mechanism of Mg/Zn-MOF inhibiting pyroptosis

**Figure 4.**
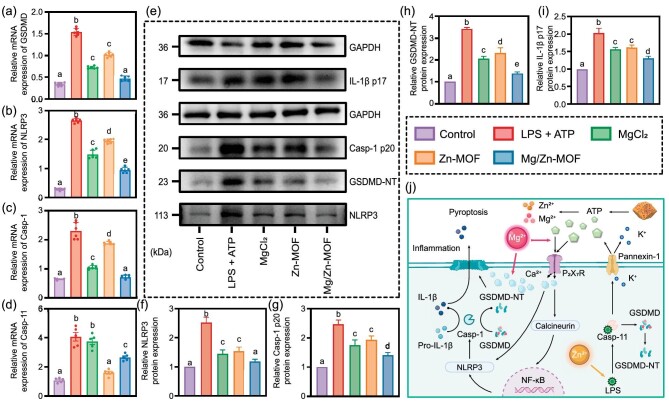
Mg/Zn-MOF inhibits the expression of pyroptosis-related proteins. (a–d) RT–qPCR results of pyroptosis-related genes (GSDMD, NLRP3, Caspase-1 and Caspase-11) (*n* = 6). (e) The levels of pyroptosis-related proteins in whole-cell lysate were detected by using Western blot. (f–i) Quantitative analysis of protein expression, where glyceraldehyde-3-phosphate dehydrogenase (GAPDH) served as the internal control. (j) Mechanism diagram of Mg/Zn-MOF inhibiting pyroptosis. (*n* = 3, *P* < 0.05, error bars indicate means ± standard deviations, and different alphabets in bar charts indicate statistically significant differences between the two groups).

Previous studies have elucidated that, within the canonical pyroptosis pathway, pattern recognition receptors directly or indirectly recruit Pro-Caspase-1 through inflammasome sensors such as NLRP3, leading to its activation and subsequent conversion into Caspase-1, which then cleaves GSDMD, generating the pore-forming GSDMD-NT [46,47]. To verify whether Mg/Zn-MOF mitigates pyroptosis via this canonical pathway, we analysed gene expressions of GSDMD, NLRP3 and Caspase-1 (Fig. 4a–c). Post LPS and ATP treatment, expressions of these genes were significantly increased compared with those of controls but decreased in the MgCl_2_, Zn-MOF and Mg/Zn-MOF groups, with the most substantial reduction observed in the Mg/Zn-MOF group. This implies an outstanding inhibition of the NLRP3/Caspase-1/GSDMD pathway by the synergistic effect of Zn^2+^ and Mg^2+^. Interestingly, the Zn-MOF group demonstrated a significant decrease in GSDMD mRNA levels, comparable to the MgCl_2_ group, but maintained higher NLRP3 and Caspase-1 expression, indicating the involvement of the other pathways in the modulation of GSDMD expression by Zn-MOF .

Considering the impact of Zn^2+^ on cellular responsiveness to LPS, it is plausible that Zn-MOF mitigates non-canonical LPS-induced pyroptosis pathways, thus reducing GSDMD expression. In the non-canonical pathway, LPS binds to Caspase-4/5 or murine homolog Caspase-11, leading to ATP and K+ release through the Pannexin-1 channel [48]. ATP activates P2 × 7R, allowing Ca^2+^ influx that not only initiates NLRP3 inflammasome assembly, but is also integral to GSDMD pore formation [17,49]. Hence, assessing Caspase-11 expression can provide insights into the non-canonical pathway. Compared with the LPS + ATP group, the Zn-MOF and Mg/Zn-MOF groups showed significantly lower Caspase-11 mRNA levels, unlike the MgCl_2_ group, indicating that Zn^2+^ effectively inhibits the Caspase-11/GSDMD pathway (Fig. 4d). Furthermore, the intracellular Ca^2+^ concentration in the LPS + ATP group was significantly reduced to varying degrees by treatments with MgCl_2_, Zn-MOF and Mg/Zn-MOF (Fig. S7). Notably, the Mg/Zn-MOF group exhibited the most pronounced reduction, almost matching the inhibitory effect of the P_2_X_7_R antagonist (A-804598). This observation suggested that Mg^2+^ and Zn^2+^ released by Mg/Zn-MOF synergistically reduce Ca^2+^ influx. To sum up, we concluded that Mg/Zn-MOF could inhibit pyroptosis via both the NLRP3/Caspase-1/GSDMD and Caspase-11/GSDMD pathways.

LPS and ATP could activate the NLRP3 inflammasome. NLRP3 triggers the assembly of inflammatory polymers, recruiting Pro-Caspase-1 into the NLRP3 inflammasome and resulting in the hydrolysis of Pro-Caspase-1 into p20 and p10 subunits, which further assemble into active Caspase-1 tetramers. GSDMD is cleaved by active Caspase-1 into GSDMD-NT, forming transmembrane pores to secret the cytokines. To further investigate the mechanism of Mg/Zn-MOF on LPS-ATP-induced pyroptosis, Western blot was conducted to analyse the pyroptosis-related proteins (NLRP3, Caspase-1 p20, GSDMD-NT and IL-1β p17). LPS-ATP stimulation obviously induced pyroptosis, as indicated by the elevated protein levels of NLRP3, Caspase-1 p20, GSDMD-NT and IL-1β p17, which were suppressed by treatment with MgCl_2_, Zn-MOF and Mg/Zn-MOF, with the most pronounced inhibition of pyroptosis-related proteins occurring in the Mg/Zn-MOF group (Fig. 4e–i and Fig. S8). As such, cells treated with Mg/Zn-MOF exhibited a reduction in damage-associated molecular pattern signaling, leading to lower NLRP3 protein expression and subsequent decreases in Caspase-1 p20 and GSDMD-NT levels, thereby diminishing transmembrane pore formation.

Interestingly, the strategy of ‘Zn^2+^ induces pyroptosis’ has been implemented in tumor therapy [50]. The seemingly paradoxical perspective actually arises from distinct strategies employed in ion-interference therapy within diverse microenvironments, underscoring the significance of Zn^2+^ regulation. While previous literature describes strategies to facilitate complete internalization of nanomaterials by tumor cells and subsequent degradation within the acidic intracellular microenvironment, leading to rapid Zn^2+^ overload and ultimately triggering pyroptosis in tumor cells, our study observes Mg/Zn-MOF decomposition within the inflammatory microenvironment, characterized by high levels of extracellular ATP. Released Zn^2+^ from nanocomposites exhibited regulatory functions extracellularly and consequently inhibited pyroptosis-related signaling pathways. Numerous studies have demonstrated that exogenous supplementation of Zn^2+^ or Mg^2+^ effectively impedes the influx of Ca^2+^ via P_2_X_7_R—a pivotal and indispensable factor in the formation of pyroptotic pores [17,51,52]. Therefore, the crux of this seemingly contradictory assertion lies in whether Zn^2+^ overload occurs intracellularly. Zn^2+^ probe (TSQ) detection results demonstrated the absence of a significant quantity of Zn^2+^ (indicated by blue fluorescence) in L929 cells incubated with Mg/Zn-MOF at the safe concentration, thereby indicating undisturbed ion balance and favorable cell viability (Fig. S12).

As illustrated in Fig. 4j, our finding suggests that Mg/Zn-MOF may target three processes to protect cells from pyroptosis. Firstly, Zn^2+^ released by Mg/Zn-MOF potentially reduces cellular responsiveness to LPS, thus curbing Caspase-11 activation. Furthermore, Zn^2+^ and Mg^2+^ together may attenuate ATP levels, disrupting the NLRP3/Caspase-1/GSDMD pathway. Lastly, Mg^2+^ acts as a physiological Ca^2+^ antagonist, influencing the membrane binding and oligomerization processes of GSDMD-NT. These combined actions underscore the efficacy of Mg/Zn-MOF in shielding gingival fibroblasts from pyroptosis *in vitro*.

### Application of Mg/Zn-MOF in an animal model of periodontitis

**Figure 5.**
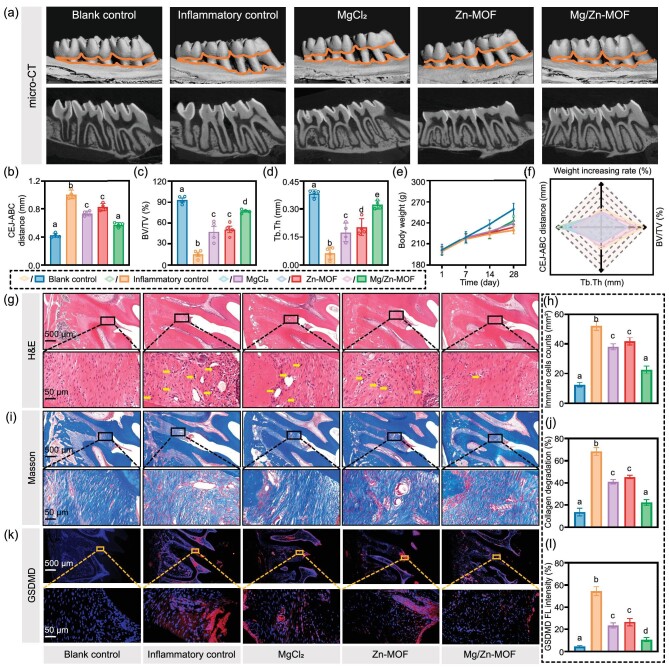
Evaluations of the *in vivo* therapeutic effect of various materials. (a) Representative micro-CT scans and 3D reconstruction models of the maxilla. (b) Distance from CEJ to ABC in mm. (c) Bone volume fraction and (d) trabecular thickness of alveolar bone around the first molar. (e) Weight data at Days 1, 7, 14 and 28 of initiation of the *in vivo* experiment. (f) Radar plot of multiple related indicators. (g) Representative H&E staining images (yellow arrows: inflammatory cells). (h) Quantitative assessment of inflammatory cells. (i) Representative Masson’s trichrome staining. (j) Quantitative assessment of collagen degradation. (k) Immunofluorescence staining of GSDMD. (l) Quantitative analysis of immunofluorescence intensity. (*n* = 4, *P* < 0.05, error bars indicate means ± standard deviations, and different alphabets in bar charts indicate statistically significant differences between the two groups).

To validate the efficacy of Mg/Zn-MOF in protecting periodontal cells from pyroptosis *in vitro*, an *in vivo* rat model of periodontitis was established (Fig. S9). Periodontitis was induced in the rats through ligation of the maxillary first molars with 0.2-mm orthodontic wire, leading to the noticeable gingival recession, redness, swelling and bleeding upon probing after 7 days. For the next 14 days, various treatments were administrated into the periodontal pockets on alternate days. Assessments of treating efficacy were performed using micro computed tomography (micro-CT) scans, 3D reconstructions and measurements of the distance between the cementoenamel junction and the alveolar bone crest (CEJ–ABC), along with hematoxylin and eosin (H&E) staining, Masson’s staining and immunofluorescence analyses. The inflammatory control group exhibited significant alveolar bone loss and resorption at the root bifurcation, confirming successful model establishment. The Mg/Zn-MOF group showed the most considerable improvement in alveolar bone preservation (Fig. 5a and b) and quantitative analyses of bone volume fraction (bone volume/total volume) and trabecular thickness (Tb.Th) supported this observation (Fig. 5c and d). These data suggest that Mg/Zn-MOF is markedly effective in restoring bone structure and density.

Weight monitoring was pivotal due to the known correlation between periodontitis and systemic health [53]. A stunted weight gain following ligation was reversed post-treatment, implying the therapeutic benefits of Mg/Zn-MOF in alleviating periodontitis-related systemic effects and reducing mortality risk (Fig. 5e). The ability of Mg/Zn-MOF to protect periodontal tissue from severe bone loss and enhance bone regeneration surpassed that of MgCl_2_ and Zn-MOF, and its role in indirectly improving growth rates suggests it also mitigates systemic damage caused by periodontitis (Fig. 5f).

H&E and Masson's trichrome staining were utilized to assess the inflammatory infiltrate and collagen integrity around the first molar. Chronic inflammation marked by dense inflammatory cell infiltrates was evident in the inflammation control group but was notably reduced after treatment, especially in the Mg/Zn-MOF group, indicating its significant anti-inflammatory effect (Fig. 5g and h). Additionally, the treatment groups showed improved collagen fiber alignment, with the Mg/Zn-MOF group displaying a pattern that was similar to that of the blank control, suggesting the protective role of Mg/Zn-MOF for gingival fibroblasts (Fig. 5i and j). Immunofluorescence staining for GSDMD protein further verified that Mg/Zn-MOF shielded gingival connective tissue from inflammatory damage and tissue destruction by inhibiting pyroptosis, with the most substantial reduction in GSDMD expression seen in the Mg/Zn-MOF group (Fig. 5k and l). These results endorse Mg/Zn-MOF as a potent nanocomposite for periodontitis treatment by alleviating the inflammatory cascade and safeguarding against tissue destruction via the regulation of pyroptosis.

### Biosafety evaluation of Mg/Zn-MOF

The biosafety of Mg/Zn-MOF was assessed using L929 cells through CCK-8 testing. The results, depicted in Fig. S10a, showed a dose-dependent decrease in cell viability with increases in the concentration of nanocomposites and the magnesium ion doping ratio, while the cell viability remained at >85% for concentrations of ≤30 μg mL^−1^, with the exception of the Mg_(30%)_/Zn-MOF group. However, at a concentration of 40 μg mL^−1^, the cell viability dropped to ∼50%. The cytotoxicity of Zn-MOF was linked to its hydrophobic nature, which depends on the surface chemical property and roughness [54,55]. Notably, at 30 μg mL^−1^, Mg_(20%)_/Zn-MOF demonstrated better biocompatibility than Mg_(30%)_/Zn-MOF, despite only slight differences in Mg^2+^ release between the two. Therefore, Zn-MOF and Mg_(20%)_/Zn-MOF at a concentration of 30 μg mL^−1^ were used in further experiments. Calcein/PI staining of L929 cells indicated that the Zn-MOF and Mg/Zn-MOF groups maintained a morphology that was comparable to that of the control group, with cells appearing short and spindle-shaped (Fig. S10b). In addition to fibroblasts, osteoblasts are integral to periodontal tissues, making it crucial to assess the biocompatibility of nanocomposites with these cells. Using the spontaneously immortalized murine calvarial cell line (MC3T3-E1), viability assays were performed through CCK-8 and calcein/PI staining. The results confirmed that concentrations of 30 μg mL^−1^ for both Zn-MOF and Mg_(20%)_/Zn-MOF are safe for osteoblasts, as illustrated in Fig. S10e and f. Furthermore, immunofluorescence staining images showed that Mg/Zn-MOF at safe concentrations did not induce pyroptosis in L929 cells (Fig. S11). Additionally, the safety assessment of nanocomposites also involves evaluating whether the ions released from the nanocomposites enter the cells in substantial amounts, potentially disrupting ion balance. TSQ fluorescence imaging revealed that the safe concentration of nanocomposites did not induce significant intracellular uptake of Zn^2+^, thereby preserving cellular ion homeostasis, as shown in Fig. S12. Intracellularly, Zn^2+^ is primarily transported from the extracellular space through Zrt- and Irt-like proteins [56].

An *in vitro* hemolysis assay further corroborated the biocompatibility of the materials, with all test materials demonstrating low hemolysis rates across the three groups (Fig. S10c). Furthermore, the animal fluorescence imaging results demonstrated the prompt metabolism and excretion of the nanocomposite within the hepatic system of mice (Fig. S13). Finally, long-term cytotoxicity was also assessed *in vivo* over a 30-day period, with no significant histopathological changes or inflammation detected in major organs (Fig. S10d). These findings collectively demonstrate that Mg_(20%)_/Zn-MOF at the tested concentration is highly biocompatible, supporting its potential application in periodontitis treatment at both the cellular and tissue levels.

## CONCLUSION

In summary, we have constructed a nanocomposite that leverages a bimetallic MOF strategy to suppress cell pyroptosis, offering a novel therapeutic approach for periodontitis. This nanocomposite demonstrated heightened ATP responsiveness, stemming from the ATP-Zn^2+^ coordination, which facilitates the targeted release of therapeutic ions. The presence of Mg/Zn-MOF downregulates GSDMD to inhibit the formation of membrane pores and consequently decrease pyroptosis, as well as the release of LDH release and IL-1β. Within this nanocomposite, Mg^2+^ and Zn^2+^ work in concert to inhibit pyroptosis via both the NLRP3/Caspase-1/GSDMD and Caspase-11/GSDMD pathways. Our findings indicate that this strategy can effectively attenuate inflammatory cell infiltration and decelerate collagen fiber breakdown, thereby preserving periodontal tissue. Thus, our research supports the clinical advancement of ion-interference therapy as a promising direction for the management and treatment of periodontitis. This warrants in-depth investigation to facilitate clinical translation.

## METHODS

### Synthesis of Mg/Zn-MOF

Zn-MOF and Mg/Zn-MOF nanocomposites were synthesized via a solvent thermal method, employing Zn(NO_3_)_2_·6H_2_O and Mg(NO_3_)_2_·6H_2_O as metal sources. Specifically, Mg/Zn-MOF was prepared by partially substituting zinc ions with magnesium at a molar ratio of 10%, 20% and 30%. For comprehensive synthesis details, please refer to the Supplementary information section.

### Characterization

Nanocrystal morphology was examined by using SEM (TECNAI F20, FEI, USA) and TEM (JEOL, JEM-2100F, Japan). Particle sizing and zeta potential were determined by DLS using the Malvern particle sizer (Malvern Corporation, England, UK). XRD patterns were recorded on an XRD instrument (Rigaku, Tokyo, Japan). UV–visible absorption spectroscopy was conducted in the wavelength range of 200–400 nm utilizing a Shimadzu UV-2550 spectrophotometer (Shimadzu Corporation, Tokyo, Japan). Elemental analysis was performed via XPS (ESCALAB 250Xi, Thermo Fisher Scientific, USA).

### *In vitro* evaluation of Mg/Zn-MOF inhibiting pyroptosis

The L929 cell line was treated with LPS and ATP to establish an *in vitro* pyroptosis model. The efficacy of Mg/Zn-MOF in inhibiting pyroptosis was gauged by using PI dye uptake, LDH and IL-1β secretion. Immunofluorescence staining was employed to ascertain the impact on pyroptosis-related proteins. Additionally, RT-qPCR and Western blot analyses were conducted to measure the expression of pyroptosis-associated genes and proteins, respectively. Further experimental details are available in the Supplementary information.

### Therapeutic efficacy of Mg/Zn-MOF in an *in vivo* model of periodontitis

Wistar rats (6 weeks old, male) were categorized into five groups (Blank control, Inflammatory control, MgCl_2_, Zn-MOF and Mg/Zn-MOF) based on the approved protocol by the Animal Experiment Ethics Inspection Committee of Jilin University (JLUKQ #KT202003050). Periodontitis was induced by ligation of the maxillary first molars. After 7 days, gingival atrophy and bleeding on probing were observed around the first molar of the rats; subsequently, various materials were administered every other day for 1 more week. Post-treatment, micro-CT scanning and histological staining (H&E and Masson's trichrome) were performed on extracted maxillae and major organs to analyse the bone mineral density and evaluate tissue recovery and inflammation. Full procedural details are outlined in the Supplementary information.

## Supplementary Material

nwae225_Supplemental_File
