# Supplementary material for: An ATP-responsive metal–organic framework against periodontitis via synergistic ion-interference-mediated pyroptosis
Source: Natl Sci Rev. 2024 Jun 26;11(8):nwae225. doi: 10.1093/nsr/nwae225 (PMC11275458; doi:10.1093/nsr/nwae225)
Supplement: nwae225_Supplemental_File [file nwae225_supplemental_file.pdf]

Supplementary Information for

An ATP-Responsive Metal-Organic Framework against Periodontitis *via* Synergistic Ion-Interference-Mediated Pyroptosis

Qijing Yang [1+], Xiaolin Sun [1+], Qihang Ding [1,2+], Manlin Qi [1], Chengyu Liu [1], Tingxuan Li [1], Fangyu Shi [1], Lin Wang [1*], Chunyan Li [1*], Jong Seung Kim [2*]

[1] Department of Prosthodontics, Jilin Provincial Engineering Laboratory of Intelligent Oral Treatment Technology, School and Hospital of Stomatology, Jilin University, Changchun, 130021

[2] Department of Chemistry, Korea University, Seoul, 02841, Republic of Korea

+These authors contribute equally to this work.

*Authors to whom correspondence should be addressed.

Prof. Jong Seung Kim, E-mail: jongskim@korea.ac.kr

Prof. Chunyan Li, Email: cyli@jlu.edu.cn

Prof. Lin Wang, Email: wanglin1982@jlu.edu.cn

This file includes:

Supplementary experimental methods

Supplementary Tables S1-S3

Supplementary Figures S1-S13 and notes

Supplementary Experimental Methods

Synthesis of Zn-MOF and Mg/Zn-MOF

The 2.5 mmol $Zn(NO_3)_2·6H_2O$ (Sigma-Aldrich, St. Louis, MO, USA) and 40 mmol 2-methylimidazole (Sigma-Aldrich, St. Louis, MO, USA) were dissolved in 20 mL and 80 mL methanol solutions (Sinopharm, Shanghai, China), respectively. Then, methanol solutions of two different solutions were mixed and stirred at 30 °C for 4 h. Afterward, the mixture was kept at 60 °C for 1 h and then centrifuged at 10,000 rpm for 15 min. The precipitate was washed with methanol solution and centrifuged again at the same speed. Finally, the centrifuged precipitate was dried in an air oven at 60 °C overnight to obtain Zn-MOF. With regard to Mg/Zn-MOF, $Mg(NO_3)_2·6H_2O$ (Sigma-Aldrich, St. Louis, MO, USA) was taken as the source of magnesium. Taking $Mg_{(10\%)}$/Zn-MOF as an example, $Mg(NO_3)_2·6H_2O$ was used to replace 2.5 mmol $Zn(NO_3)_2·6H_2O$ with 10% molar ratio. That is to say, 0.25mmol $Mg(NO_3)_2·6H_2O$ and 2.25 mmol $Zn(NO_3)_2·6H_2O$ were respectively added into 10 mL methanol solution to dissolve, thus obtaining the solution with [Mg/(Mg+Zn)] equal to 10% molar ratio. The following steps were the same as those for the synthesis of prinstine Zn-MOF.

Density functional theory (DFT)

The calculations were carried out using periodic DFT method in the generalized gradient approximation (GGA) of Perdew-Burde-Erzerhof (PBE) with a plane wave basis set in CASTEP. The plane wave cutoff energy was set to be 435.4 eV, the convergence threshold was set as $2×10^{-6}$ eV/atom, maximum force magnitude that remained on each atom had been limited to 0.05 eV/Å.

Cell culture

The mouse fibroblast cells (L929) , obtained from the Institute of Biochemistry and Cell Biology, the Chinese Academy of Sciences (Shanghai, China), cultured in Dulbecco's Modified Eagle Medium (DMEM, Gibco, Grand Island, NY, USA) supplemented with 10% fetal bovine serum (FBS, Gibco, Grand Island, NY, USA) and 1% penicillin–streptomycin (100 U mL^{-1} penicillin and 100 g mL^{-1} streptomycin) and incubated at 37 °C in an atmosphere with 5% CO_2 and 100% humidity. The spontaneously immortalized murine calvarial cell line (MC3T3-E1) cultured in alpha-MEM (alpha-MEM, Gibco, Grand Island, NY, USA) supplemented with 10% fetal bovine serum (FBS, Gibco, Grand Island, NY, USA) and 1% penicillin–streptomycin (100 U

mL^{-1} penicillin and 100 g mL^{-1} streptomycin) and incubated at 37 °C in an atmosphere with 5% CO_2 and 100% humidity.

Cell model establishment and treatment

According to the characteristics of the periodontal immune microenvironment, L929 cells were stimulated with LPS + ATP to induce pyroptosis. Following stimulation with LPS (1 μg mL^{-1}), ATP (5mM) was added and incubated for 1 h. To investigate the protective effect of each material, the cells were pretreated with $MgCl_2$ (2.7625 mmol), Zn-MOF (30 μg mL^{-1}) or Mg/Zn-MOF (30 μg mL^{-1}) for 12 h, respectively. The same volume of cell culture medium was added in the LPS + ATP group. The blank control group received an equivalent volume of cell culture medium without stimulation.

Cytocompatibility assay for Mg/Zn-MOF

5×10^{3} fibroblasts were placed in each well of 96-well plates and were cultured overnight to adhere to the wall. Each group of Zn-MOF and Mg/Zn-MOF was prepared into four concentrations of 10 μg mL^{-1} to 40 μg mL^{-1} media, and the L929 and MC3T3-E1 cells were cultured in above the media for 24 h. Then, cell viability was determined by measuring the absorbance value at 450 nm using a Cell Counting Kit-8 (CCK-8, NCM, China) on a microplate reader (Bio-Tek, Winooski, VT, USA). Each experimental group setted six paralleled wells. By calculating the survival rate of fibroblasts, the 30 μg mL^{-1} of Zn-MOF or Mg/Zn-MOF were finally selected as the optimal concentratio.

In addition, The L929 and MC3T3-E1 cells were seeded in a 24-well plate with the dosage of 2×10^{4} cells at 37 °C for 24 h. The culture wells within well-adhesive cells were treated with either Zn-MOF or Mg/Zn-MOF at a concentration of 30 μg mL^{-1}. Group with equivalent normal culture medium addition was served as blank control. The L929 cells were stained using the Calcein/PI Cell Viability/Cytotoxicity Assay Kit (Beyotime, Shanghai, China). After incubation at 37 °C for 30 minutes in the absence of light, the staining effect and cell morphology were observed under a fluorescence microscope (TH4-200, U-RFL-T, Olympus, Tokyo). Six wells were tested for each group.

In order to obverse the biosafety of nanomedicine, BALB/c mice were injected with equivalent nanodrug (0.2 mL per site) and their main organs were acquired two month later to explore pathological changes (n = 6). The animal experiment followed a protocol that was approved by Jilin University (JLUKQ #202012025).

To evaluate the hemolytic activity of the nanomaterial, fresh rat blood was collected in anticoagulant tubes containing EDTA, vigorously mixed to ensure uniformity, and subsequently centrifuged at 3000 rpm for 15 minutes to isolate red blood cells (RBCs). Then 1 mL of test solution (Zn-MOF, Mg/Zn-MOF, $MgCl_2$, PBS and ddH_2O) was added to 20 μL red blood cells. PBS was added as negative control and ddH_2O as positive control. After standing at 37 °C for 2 h, the suspension was centrifuged again. The supernatant from each centrifugal tube was used to analyze the hemoglobin release using a microplate reader (BioTek, Vermont, USA) at 540 nm. Three samples were used for each group. Percentage hemolysis was calculated using the following formula:

$$\text{Hemolysis (\%)} = \frac{\text{OD(sample)} - \text{OD(negative control)}}{\text{OD(positive control)} - \text{OD(negative control)}} \times 100\%$$

Hoechst 33342/PI fluorescent staining

L929 cells were cultured in a 24-well plate until the cell confluence reached 80%. The cells were treated with different agents according to the vary experimental groups. Afterward, a Hoechst 33342/PI Double Stain Kit (Yeasen, Shanghai, China) was used according to the manufacturer's instructions. In brief, the L929 cells were stained with 5 μL Hoechst 33342 staining solution and 5 μl PI staining solution for 20 min at 4 ℃. Subsequently, stained cells were immediately observed under a fluorescence microscope (TH4-200, U-RFL-T, Olympus, Tokyo).

Lactate dehydrogenase (LDH) release assay

L929 cells were seeded in a 6-well plate at a number of 2×10^5. The cells were subjected to distinct treatments based on the specific experimental groups. The LDH activity in the cell supernatant was measured using an LDH assay kit (Nanjing Jiancheng Institute of Biological Engineering, Nanjing, China) following the manufacturer's instructions. The detection reagents were mixed with the samples to be tested in each group according to the specified protocols and incubated at 37 ℃. Subsequently, they were transferred into a 96-well

plate and allowed to stand at room temperature for 5 minutes. Then, absorbance values were subsequently measured at 450nm using a microplate reader. LDH activity was calculated using the following formula:

$$LDH\ Activity\ (\frac{U}{gprot}) = \frac{OD(sample) - OD(control)}{OD(standard) - OD(blank)} \times C(standard) \div Cpr$$

C(standard): The concentration of the standard solution (0.2 μmol mL^{-1}).

Cpr: The protein concentration of the sample (gprot mL^{-1}).

Enzyme-linked immunosorbent assay (ELISA)

The cell culture medium from each group was collected, followed by centrifugation (500 g, 5 min), and the resulting supernatant was used as the test sample. The quantification of IL-1β was performed using a Mouse IL-1β Double Antibody Sandwich ELISA Kit (Proteintech, Illinois, USA) in accordance with the manufacturer's instructions.

Quantitative Real-time PCR (RT-qPCR)

L929 cells were seeded into 6-well plates with a number of 2×10^5 and treated according to the experimental protocol. Total RNA was extracted by TRIzol (Thermo Scientific, Waltham, MA, USA) according to manufacturer instruction and prepared for cDNA via reverse transcription utilizing the PrimeScript RT Reagent Kit (Takara, Kusatsu, Shiga, Japan). Through the application of SYBR Premix Ex Taq II kits (Takara, Kusatsu, Shiga, Japan), the gene expression factors relevant to pyroptosis (NLRP3, Caspase-1, Caspase-11, GSDMD and IL-1β) were detected by QPCR Mx3005P system (Agilent, Santa Clara, California, USA). The primer sequences used for qPCR were described in Table S2. The relative levels of disparate genes expression were calculated by $2^{-\Delta\Delta Ct}$ method and normalized to housekeeping gene β-actin. The Ct value of blank control group was served as calibrators. All experiments were repeated six times.

Western blot

The protein levels of NLRP3, Caspase-1, IL-1β and GSDMD were quantitively investigated via western blot. The lysis buffer was prepared by mixing PMSF (Beyotime, Shanghai, China) and RIPA (Beyotime, Shanghai, China) at a ratio of 1:100. Subsequently, the cells were lysed on ice for 30 minutes followed by centrifugation at 14,000 rpm for 15 minutes to obtain total protein. Protein quantification was performed using

the BCA Protein Quantification Kit (NCM, Suzhou, China). Protein samples were separated by SDS-PAGE electrophoresis and then transferred to PVDF membrane (Millipore, Merck, Germany). Among them, IL-1β was resolved using a high concentration SDS-PAGE gel (20%) due to its lower molecular weight, while other proteins were separated by a conventional 10% SDS-PAGE gel. After overnight incubation at 4 °C with the primary antibody, the membrane was subsequently exposed to the appropriate secondary horseradish peroxidase (HRP)-conjugated antibody (Beyotime, Shanghai, China) for a duration of 1 hour. The primary antibodies used included Anti-NLRP3 antibody (ab263899, Abcam, Shanghai, China), Anti-Caspase-1 antibody (sc-392736, Santa Cruz Biotechnology, Shanghai, China), Anti-IL-1β antibody (sc-52012, Santa Cruz Biotechnology, Shanghai, China), Anti-GSDMD antibody (WL05686, Wanleibio, Shenyang, China) and Anti-GAPDH antibody (ab8245, Abcam, Shanghai, China). After treatment with the ECL kit (NCM, Suzhou, China), proteins were visualized and protein expression was evaluated using a chemiluminescence image analysis system (JUNYI, Beijing, China). GAPDH was used as internal control.

Immunofluorescence (IF)

L929 cells were seeded in a 24-well plate at a number of 4×10^4 and treated as per the experimental protocol. Following fixation of cells with 4% paraformaldehyde fix solution (Beyotime, Shanghai, China) for 10 minutes, permeabilization was achieved using 0.2% Triton X-100 (Beyotime, Shanghai, China) for 2 minutes, followed by a blocking step in 5% Bovine Serum Albumin (BSA, Solarbio, Beijing, China) for 1 hour to prevent non-specific protein-protein interactions. Subsequently, the cells were incubated overnight at 4 °C with the anti-GSDMD primary antibody (WL05686, Wanleibio, Shenyang, China), and then exposed to anti-Rabbit IgG H&L (Alexa Fluor 647, ab150079, Abcam, Shanghai, China) at room temperature for 1 hour. Finally, DAPI staining was performed to visualize the nuclei. GSDMD-positive cells were recorded by fluorescence microscopy (TH4-200, U-RFL-T, Olympus, Tokyo).

Stablishment of rat periodontitis model

Wistar rats (6 weeks old, male) were obtained and used under protocols approved by the Committee for Animal Research of Jilin University (JLUKQ #KT202003050). All rats were kept and operated in accordance with the guidelines for the care and use of laboratory animals in China. Initially, Wistar rats were immobilized

on a rodent anesthesia machine, and anesthesia was induced using isoflurane (RWD, Shenzhen, China) in accordance with the rats' body weight. To induce ligature-induced periodontitis, orthodontic ligature wires (0.2 mm diameter) were tied around the maxillary first molar. After seven days, the periodontitis model was established. Various materials including $MgCl_2$, Zn-MOF and Mg/Zn-MOF were then administrated in situ with a dosage of 0.1 mL per rat every alternate day for a duration of 14 days. The inflammatory Control group was injected equal volume of saline instead. The blank control group was given saline without ligation for the whole process.

Micro-computed tomography (micro-CT)

The hard tissues containing maxillary and teeth were scanned with a micro-computed tomography scanner (Scanco Medical AG, Bassersdorf, Switzerland) to assess bone loss. The vertical distance between the alveolar bone crest (ABC) and the cementoenamel junction (CEJ) in the maxillary first molar was calculated as a measure of bone loss following 3D CT image reconstruction.

Histological evaluation

After treatment, all rats were sacrificed, the hard tissues of maxillary and teeth were excised, fixed in 4% PFA for 48 hours, decalcified and embedded in paraffin. The immunofluorescence staining, H&E staining and Masson’s trichrome assay were conducted to further evaluate the treatment effect. The slides were examined by a light microscope (Olympus) at 100 × magnification to identify inflammatory areas, and at 400 × magnification to quantitative analysis.

Statistical analysis

All data were presented as mean ± standard deviation. Statistically significant differences were indicated by distinct letters on the bar charts. Analyses was performed with SPSS 19.0 software (SPSS, Chicago, IL, USA), employing one-way ANOVA followed by Tukey's post-HSD test, with p value <0.05 denoting statistical significance.

Supplementary Tables

Table S1. Lattice parameters calculated by DFT.

Sample	a	b	c
Zn-MOF	17.63	17.63	17.63
$Mg_{(10\%)}$/Zn-MOF	17.00	17.03	17.06
$Mg_{(20\%)}$/Zn-MOF	16.91	16.91	16.95
$Mg_{(30\%)}$/Zn-MOF	17.11	16.93	17.15

Table S2. Actual Mg^{2+} concentration released and actual Mg content in Mg/Zn-MOF.

Sample	Actual Mg release (ppm)	Actual Mg content (%) [Mg/(Mg+Zn)]
$Mg_{(10\%)}$/Zn-MOF	45.6	1.88
$Mg_{(20\%)}$/Zn-MOF	66.3	2.73
$Mg_{(30\%)}$/Zn-MOF	69.8	2.87

Table S2. Primer sequences used in this study.

Gene	Forward Sequence (5' to 3')	Reverse Sequence (5' to 3')
β-actin	CATCCGTAAAGACCTCTAGCCAAC	ATGGAGCCACCGATCCACA
IL-1β	TCCAGGATGAGGACATGAGCAC	GAACGTCACACACCAGCAGGTTA
GSDMD	AGTGCTCCAGAACCAGAACC	CCTTCTCCCATGCCAGAACC
Caspase-1	GGCACATTTCCAGGACTGACTG	GCAAGACGTGTACGAGTGGTTG
NLRP3	TCACAACTCGCCCAAGGAGGAA	AAGAGACCACGGCAGAAGCTAG
Caspase-11	GCTACGATGTGGTGGTGAAAGAG	GTGCTGTCTGATGTCTGGTGTTC

Supplementary Figures

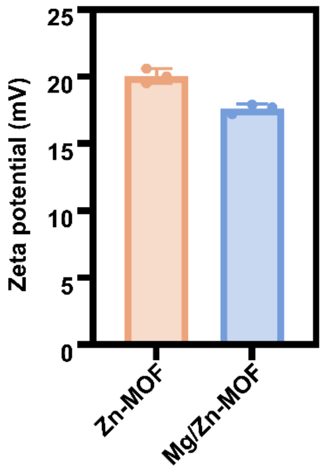

Figure S1. Zeta potential of Zn-MOF and Mg/Zn-MOF.

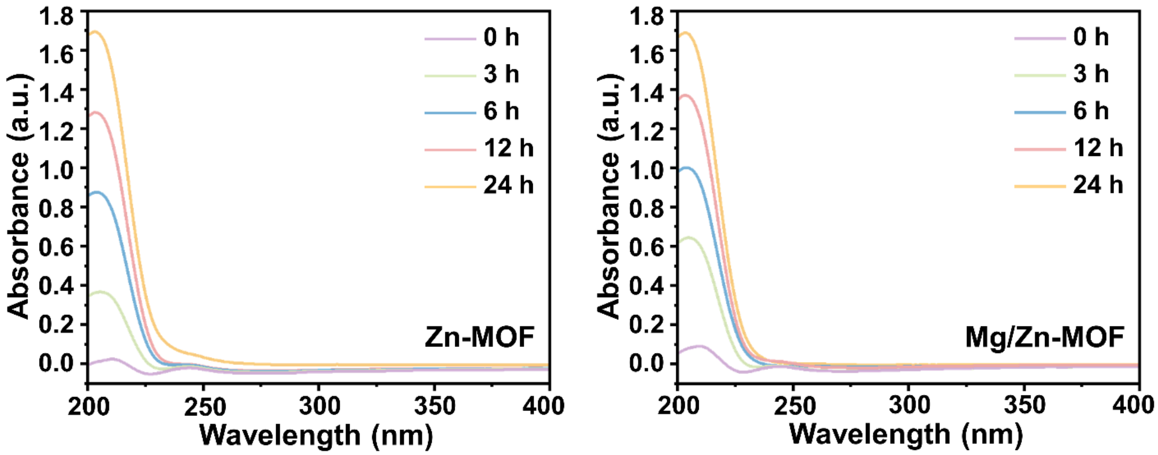

Figure S2. UV-Vis spectra of nanocomposites saline solution after immersion for various durations. To explore the release of nanocomposites in physiological environment, the Zn-MOF and Mg/Zn-MOF were placed in a dialysis bag with a current carrying molecular weight of 100 Da, and then placed in saline solution to observe the continuous release of 2-methylimidazole groups (no saline solution was replaced during this process). The dialysis bag with current carrying molecular weight of 100 Da was selected due to the molecular weight of 2-

methylimidazole groups was less than 100 Da and the molecular weight of NPs or nanocomposites was greater than 200 Da. The UV absorption peak of imidazole group gradually increased with the increase of immersion time in each experimental group. More interesting, in the soaking time more than 24 h, the imidazole group still released, suggesting that the Mg/Zn-MOF nanocomposites have excellent stability in physiological environment.

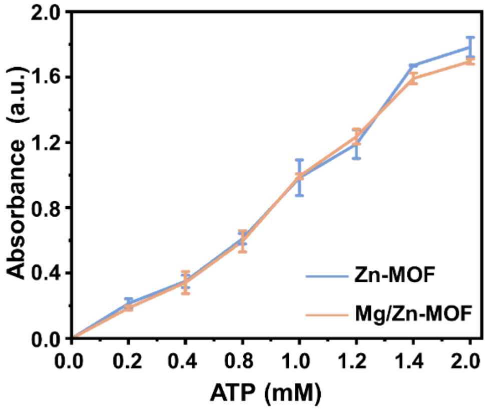

Figure S3. The correlation between nanoparticles decomposition and ATP concentration. The UV absorption peak at 207 nm served as a monitoring index.

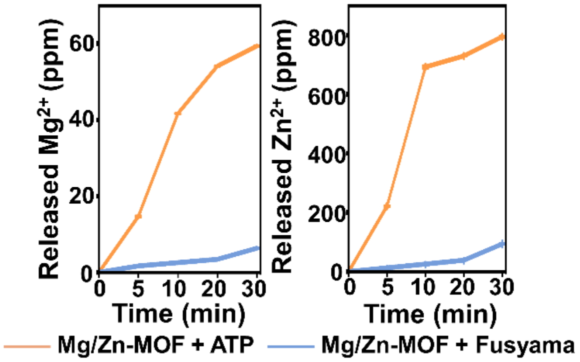

Figure S4. Levels of Zn^{2+} and Mg^{2+} released from Mg/Zn-MOF in artificial saliva and 5mM ATP (T = 37°C).

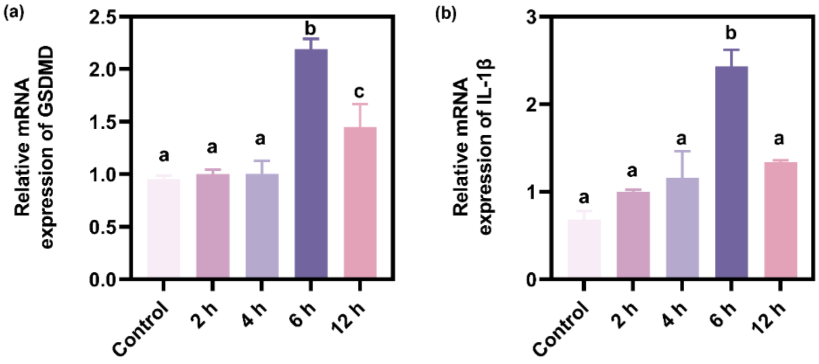

Figure S5. Expression of pyroptosis-related genes in response to diverse durations of LPS stimulation. In order to establish a more effective L929 cell pyroptosis model, it was essential to determine the optimal duration of LPS stimulation (1 μg mL^{-1}) prior to ATP induction. The mRNA expression levels of GSDMD and IL-1β were detected following treatment with 1 μg/mL LPS for 2, 4, 6, and 12 hours and subsequently stimulated with 5 mM ATP for 1 hour. (a) RT-qPCR analysis of the relative mRNA level of GSDMD. (b) The mRNA expression of IL-1β. (n = 6, $p < 0.05$, error bars indicate means ± standard deviations, different alphabets in bar charts indicate statistically significant differences between the two groups).

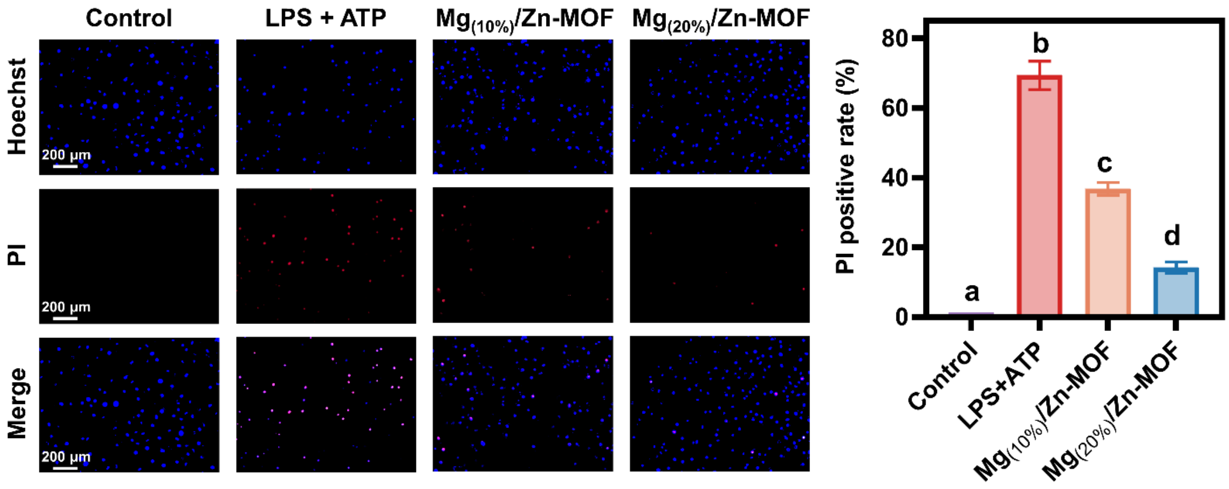

Figure S6. Representative images of Hoechst 33342 (blue) and PI (red) staining of L929 cells after different pretreatments.

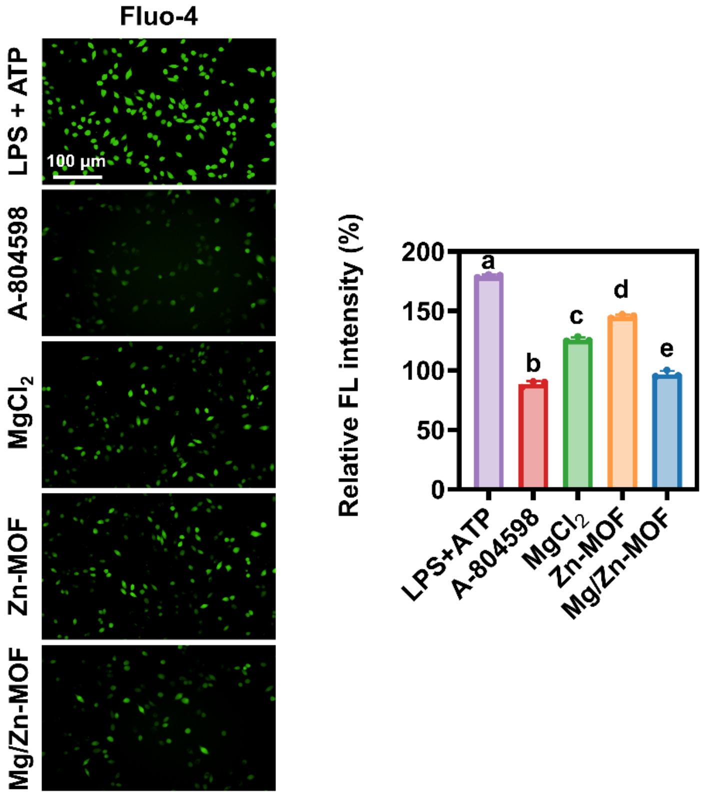

Figure S7. The fluorescence images and relative fluorescence intensity of intracellular Ca^{2+} after treatment with different materials measured by Fluo-4 Ca^{2+} detection kit. (n = 3, $p < 0.05$, error bars indicate means ± standard deviations, different alphabets in bar charts indicate statistically significant differences between the two groups).

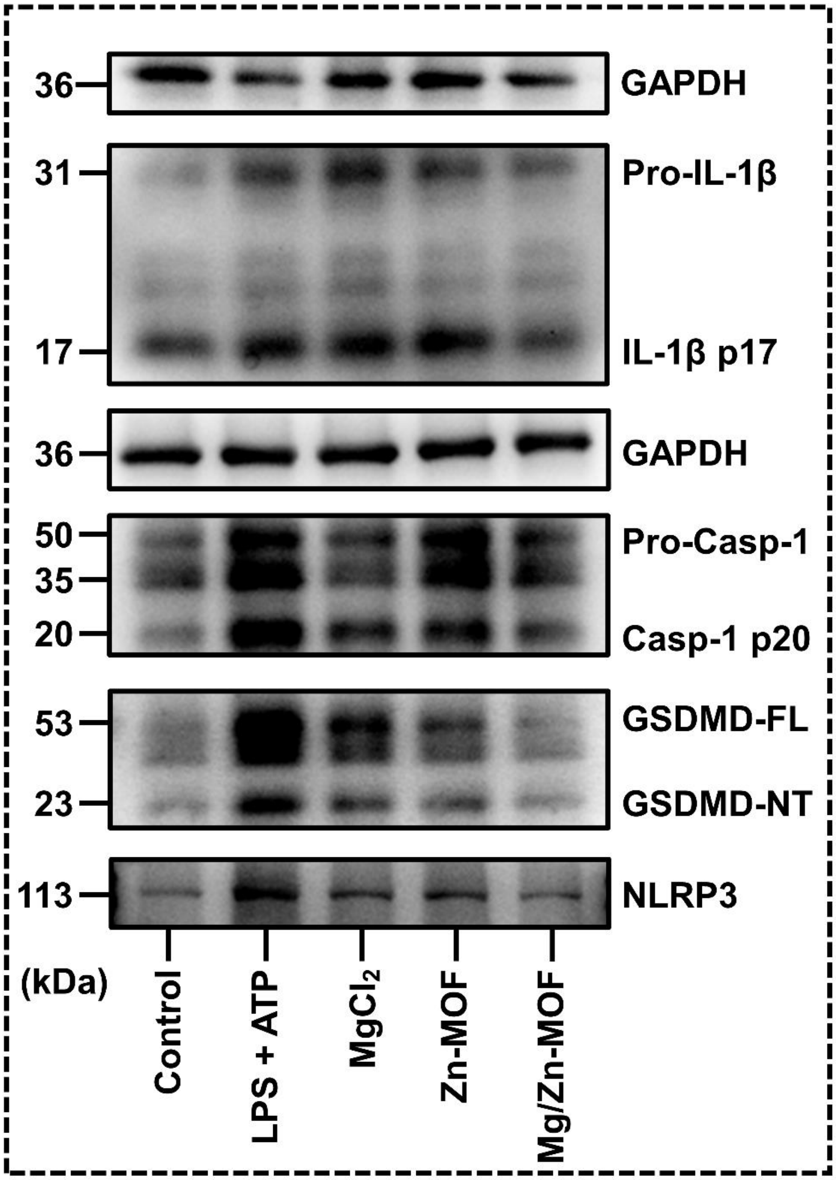

Figure S8. Immunoblot map of pyroptosis-related proteins, including precursors and spliceosomes.

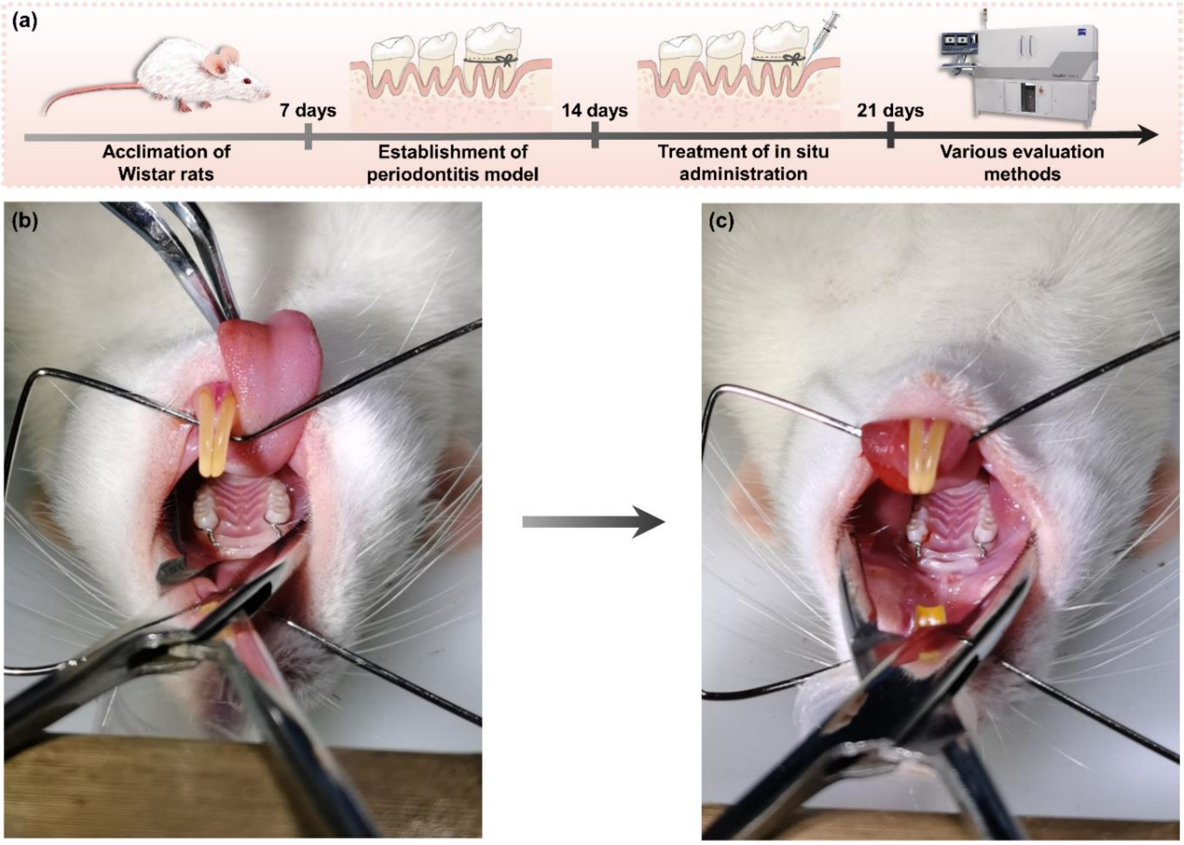

Figure S9. (a) Schematic diagram of *in vivo* experiments modeling and treatment. (b) Photograph of orthodontic ligature wires (0.2 mm diameter) fixing on the first molar during periodontitis modeling. (c) Intraoral photograph taken after successful periodontitis modeling, showing accumulation of soft plaque, gingival retraction and bleeding.

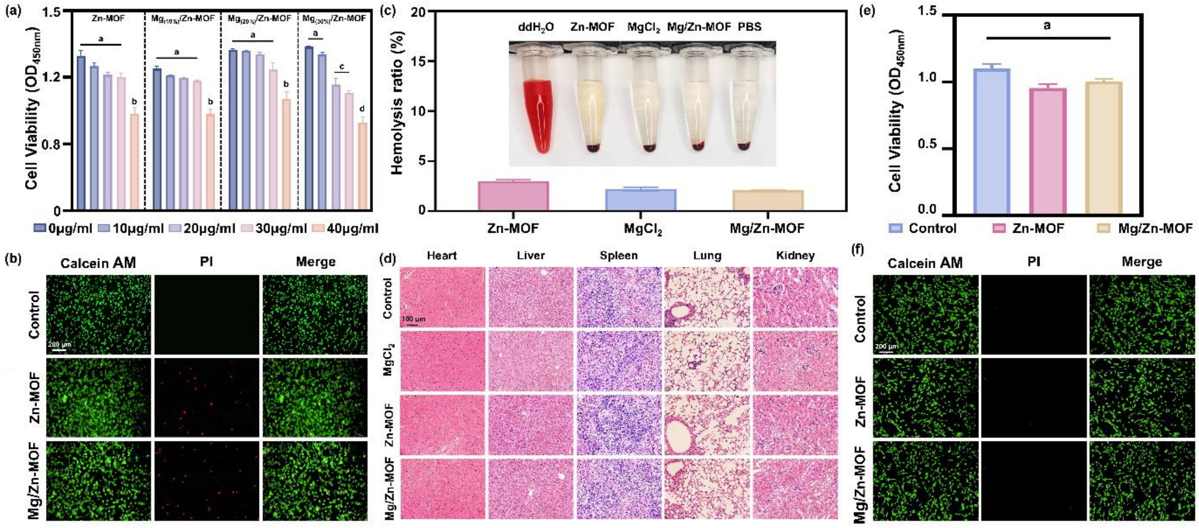

Figure S10. Biosafety assay of nanocomposites. (a)Viability of the L929 fibroblast incubated with 10-40 μg mL^{-1} Zn-MOF or Mg/Zn-MOF for 24 h. (b) Representative images of L929 incubated with 30 μg mL^{-1} NPs. Living cells stained in green by Calcein-AM, and dead cells stained in red by PI. (c) Photograph and analysis of erythrocyte hemolysis in rats in the presence of $MgCl_2$, Zn-MOF or Mg/Zn-MOF. (d) HE staining sections of main organs harvested from BALB/c mice with the treatment of subgingival injection of nanoparticles. (e) Viability of MC3T3-E1 cells incubated with 30 μg mL^{-1} Zn-MOF or Mg/Zn-MOF for 24 h. (f) Representative images of MC3T3-E1 cells incubated with 30 μg mL^{-1} nanoparticles. Living cells stained in green by Calcein-AM, and dead cells stained in red by PI. (n = 6, $p < 0.05$, error bars indicate means ± standard deviations, different alphabets in bar charts indicate statistically significant differences between the two groups).

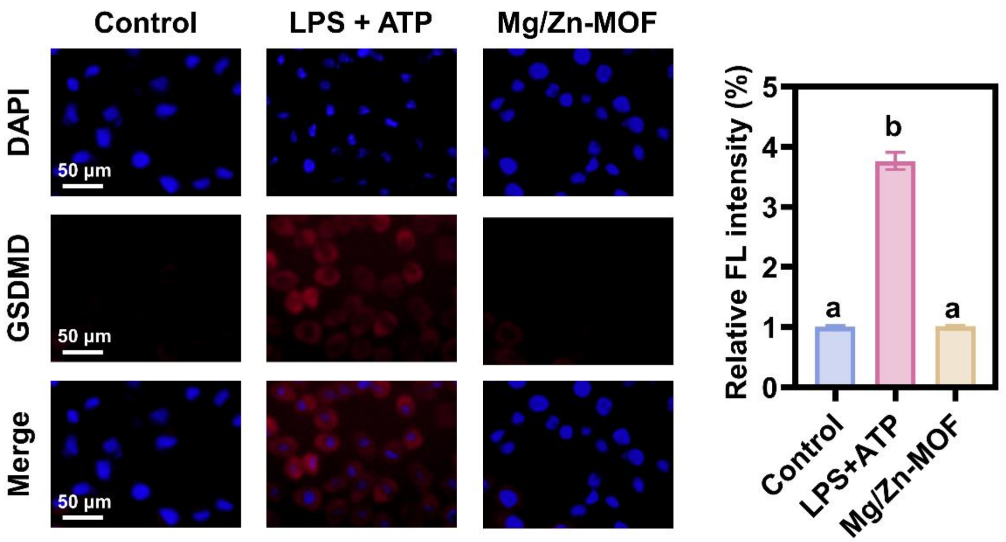

Figure S11. Representative immunofluorescence images and quantitative analysis of GSDMD expression in L929 cells after 24 hours of incubation with Mg/Zn-MOF (30 μg mL^{-1}).

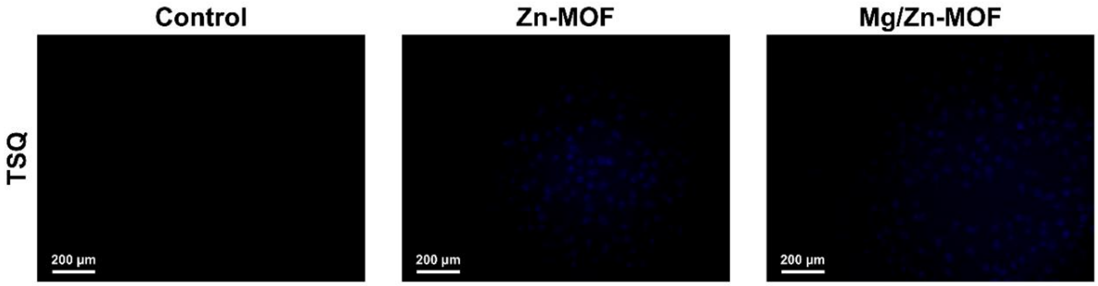

Figure S12. Intracellular Zn^{2+} ions detected by the Zn^{2+} ion probe [N-(6-Methoxy-8-quinolyl)-p-toluenesulfonamide] (TSQ).

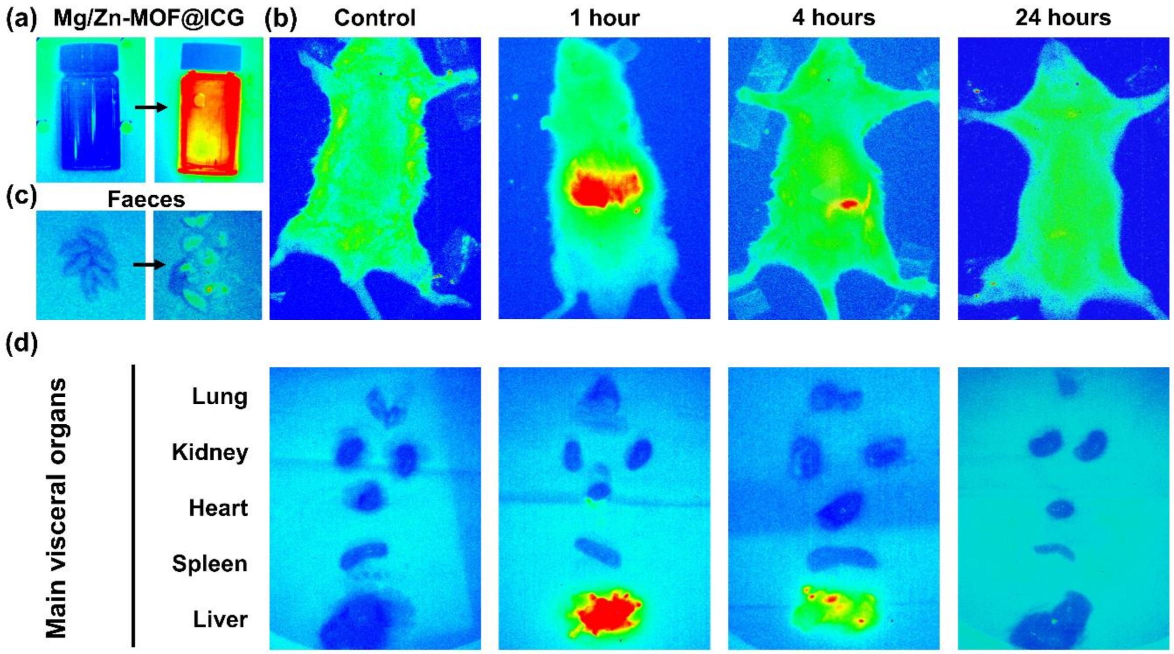

Figure S13. Animal fluorescence imaging. (a) Fluorescence images of Mg/Zn-MOF@ICG. During the stirring of Mg/Zn-MOF (left) synthesis, 5mL of ICG methanol solution (3.6 mM) was added to prepare Mg/Zn-MOF@ICG (right). (b) Animal imaging of Mg/Zn-MOF@ICG *in vivo* at various time points. (c) Fluorescent images of the metabolite of the mice treated with Mg/Zn-MOF@ICG (right) and that of the control mice (left), respectively. (d) Organ images of Mg/Zn-MOF@ICG *in vivo* at various time points.
